# Self-medication Among Myofascial Pain Patients: A Preliminary Study

**DOI:** 10.2174/1874210601812010347

**Published:** 2018-04-30

**Authors:** Gabriel Pires Pastore, Douglas Rangel Goulart, Patrícia Radaic Pastore, Alexandre Javaroni Prati, Márcio de Moraes

**Affiliations:** 1 Department of Oral and Maxillofacial Surgery, Paulista University - UNIP, São Paulo, Brazil; 2 Institute of Education and Research - IEP / Sírio Libanês Hospital, São Paulo, Brazil; 3 Piracicaba Dental School, State University of Campinas, Piracicaba, Brazil; 4 Department of Dentistry, UNIEURO University Center, Brasília, Brazil; 5 Instituto de Ensino e Pesquisa - IEP / Hospital Sírio Libanês, São Paulo, Brazil; 6 Department of Oral Diagnosis, Oral and Maxillofacial Surgery Division, State University of Campinas, Piracicaba Dental School, Piracicaba, Brazil

**Keywords:** Analgesics, Facial Pain, Fonseca`s Anamnestic Index (FAI), Self-medication, Temporomandibular Joint Disorders, Temporomandibular Joint

## Abstract

**Background:**

Self-medication has been reported as an option which people choose to relieve the suffering of conditions that cause pain, however, this could delay the correct diagnosis and therapy.

**Objective:**

The aim of the present study was to determine the prevalence of self-medication among patients with Temporomandibular Disorder (TMD), and to analyze correlations with the severity of the disease.

**Methods:**

A prospective study was conducted with patients who had been diagnosed with TMD. The patients were submitted to anamnesis and a physical examination. This research also used the Fonseca`s Anamnestic Index (FAI) and a questionnaire that was developed specifically for this study, containing questions related to the first health professional contacted and self-medication. The data were analyzed using comparative and correlative analysis (Version 18.0 of SPSS software), with the level of significance set at *p*<0.05.

**Results:**

Thirty-four patients were included, with a prevalence of females (91.2%) and a mean age of 39.76 years. Half of the patients claimed to have chosen their own medications at time, especially analgesics. Sodium dipyrone was used by 12 of the participants. Dentists were the most commonly contacted health professionals (55.5%). No correlation was found between self-medication and the severity of TMD according to the FAI. Furthermore, the time period between the onset of symptoms and the first consultation was not affected by self-medication.

**Conclusion:**

Self-medication seems to be highly prevalent among patients with TMD, although this practice does not seem to alter the severity of the disease.

## 1 INTRODUCTION

In most societies, a person who is suffering from physical and/or emotional discomfort has several options available to them. One of these options is self-medication, which can be defined as the use of medicines to treat disorders (self-diagnosed) or symptoms. One of the greatest problems with this practice is the absence of a clinical assessment by a qualified health professional, which can lead to delays in the diagnosis and adequate treatment, health risks, the inadequate use of medication, interaction with other prescribed medications and unnecessary spending [1].

The use of medication (whether prescribed or not) has become more common as a form of therapy to reduce morbidity and improve the quality of life of many individuals. A part of this group are patients who suffer from conditions that generate chronic pain. It is believed that 12% of these people are affected by TMD and 5% of this group exhibit severe symptoms that require treatment [2].

TMD is a term that is applied to functional abnormalities related to the Temporomandibular Joint (TMJ) and associated masticatory structures. The etiology of TMD is multifactorial, including trauma, malocclusion, parafunctional habits, muscular abnormalities, emotional stress, anxiety, postural abnormalities and rheumatic conditions [3]. The treatment of this disorder is based on a precise diagnosis of the location of the abnormality, which is usually divided into joint and non-joint disorders [4].

A conservative or surgical treatment plan is established for each diagnosis. The literature contains therapeutic approaches involving medication, occlusal appliance, physiotherapy, acupuncture, open surgery on the TMJ and arthroscopy, among others [4]. The most common forms of medication for this condition are analgesics, anti-inflammatories and muscle relaxants [5].

Afolabi **et al*.* (2010) [1] conducted a study in an attempt to determine the proportion of patients who seek dental services and self-medicate. In total, 536 patients were interviewed and of these, 42% admitted using medication without a prescription. The medication they selected was usually used in isolation (56.4%). They favored the use of analgesics (50.1%) and antibiotics (30.4%), for at least one week (45.8%). The general perception was that the use of these drugs would save them time and money (22.2%).

Patients with TMD exhibit symptoms such as headache and tinnitus, which lead them to seek professional help from neurologists, otolaryngologists and physiotherapists. A number of patients receive their first treatment when the TMD is already severe, due to the delay in seeking specialized help. No previous studies were identified with the specific aim of assessing the prevalence of self-medication among patients with TMD, although some have dealt with this subject [6, 7]. The aim of the present study was to identify the prevalence of self-medication among patients with temporomandibular disorder and to correlate this self-medication with the severity of the symptoms and the time period that elapsed before the initial consultation.

## 2 MATERIALS AND METHODS

A prospective study was conducted with patients complaining of pain and TMD in a dental clinic in the Paulista University (São Paulo, Brazil). The following inclusion criteria were applied: patients with myofascial pain according to clinical examination, patients who appeared at the institution for the first time; male and female individuals; aged between 18 and 80 years; with a minimum classification of mild TMD according to the Fonseca`s Anamestic Index (FAI) [8]. The following exclusion criteria were applied: Patients with chronic systemic diseases and generalized pain, including fibromyalgia and rheumatoid arthritis; patients who did not exhibit clinical signs and symptoms of TMD and patients who had undergone a previous surgical procedure on the TMJ.

Two strategies were used in sample selection in order to reduce bias: patients with no TMD signs and those with TMD signs related to joint component (Internal derangements of TMJ) were excluded. The second criteria were based on muscle sensitivity by palpation (Pressure Pain Threshold), which was performed by a single specialist. Bilateral palpations were performed on the masseter muscles (superficial and deep part), temporal (anterior, middle and posterior division), hyoid-style region and lateral pterygoid muscle. The responses to the palpations were classified into four grades: Absence of pain, slight discomfort, pain and pain accompanied by scape with the face. We selected patients who presented the “pain” response in at least in two points of the palpation.

The FAI was used to assess the severity of the TMD [8]. The FAI was developed in Brazilian Portuguese to assess the severity of TMD, based on signs and symptoms. The index in made up of ten items with three response options: “yes” (10 points), “sometimes” (5 points) and “no” (0 points). The score is determined by the sum of the points of all items and is classified as follows: absence of signs and symptoms of TMD (0-15 points); mild TMD (25-45 points); moderate TMD (50-65 points) and severe TMD (70-100 points). The English version of this questionnaire was reported by Campos **et al*.* (2009) [9] and is display in Chart **1**.

A questionnaire developed specifically for this research was also used. It contained questions related to the onset of symptoms, the first contact with a health professional and self-medication (Chart **2**). The present study was approved by the Research Ethics Committee of the Faculty of Dentistry of the UNIP.

### 2.1 Statistical Analysis

The data were analyzed using descriptive and correlational statistics, with the support of SPSS software (v. 18.0) for Windows (SPSS Inc, Chicago, IL). The Chi-square test was used to assess the relationship between the variables. The Kruskal-Wallis test was performed to compare the time period that had elapsed between the onset of symptoms and the initial consultation related to the severity of TMD. The Mann–Whitney U test was performed to compare the time period that had elapsed between the onset of symptoms and the initial consultation according to the groups of patients that report self –medication and not. The results were considered statistically significant for *p*<0.05.

## 3 RESULTS

The present study assessed 34 patients, with a prevalence of women (31/34; 91.2%), a mean (SD) age of 39.76 (14.86) years. Most of the patients had completed high school (15/34; 44.1%), followed by third level education (13/34; 38.2%) and primary education (6/34; 17.6%). Six categories were identified for the main pain complaint: pain in the TMJ (11/34; 32.4%); headaches (n= 10/34; 29.4%); difficulty while chewing (4/34; 11.8%); clicking and crackling noises in the TMJ (4/34; 11.8%); bruxism and teeth clenching (4/34; 11.8%); and tinnitus (1/34; 2.9%). Table **1** shows the distribution of sample according to variables studied.

Concerning the first health professional contacted after the onset of symptoms, a prevalence was recorded for dentists (19/34; 55.9%), followed by otolaryngologists (8/34; 23.5%), general doctors (6/34; 17.6%) and neurologists (1/34; 2.9%). The time period that elapsed between the onset of the symptoms and the first consultation ranged from 15 days to 4 years, with a mean (SD) value of 13.29 (14.88) months. Half of the patients (n=17) claimed to have self-medicated prior to seeking professional help. Most of this group used analgesics (n=15), particularly sodium dipyrone (n=12) and paracetamol (n=3), although the use of non-steroidal anti-inflammatories (n=5) and muscle relaxants (n=1) was also reported. Most of the patients used medication without an indication from another person (n=14), although two patients based their medication on the recommendations of a pharmacist and another asked a family member. In the sample, 15 patients exhibited systemic diseases. In total, 61.8% (21/34) of the patients currently take medication and of these, 90.5% (19/21) of the drugs were prescribed by a health professional. Six patients in the sample claimed to suffer from or had been diagnosed with, depression.

No statistical difference was recorded between self-medication and the level of education (X^2^=0.810; *p*=0.667) or gender (X^2^=0.366; *p*=0.545). Regarding the main complaint of patients and the health professional contacted, a dentist was most often contacted for complaints related to chewing difficulties (n=3), noises in the TMJ (n=3) and parafunction (n=4). For pain in the TMJ however, the distribution was similar among dentists (n=6) and otolaryngologists (n=4). A significant proportion of patients contacted a general practitioner for headaches (n=5).

When using the Fonseca anamnestic index, the sample exhibited an even distribution among the levels of severity: mild (n=10); moderate (n=14); and severe (n=10). No statistically significant differences were found between the presence of severe TMD and self-medication (X^2^=0.56; *p*=0.452). The mean time period that had elapsed between the onset of symptoms and the initial consultation was longer among patients with severe TMD (17.13 months) than among those with moderate TMD (9.63 months) or mild TMD (14.75 months), although there were no statistically significant differences between the groups (Kruskal-Wallis, X^2^= 2,23; *p*=0.327). No differences were found regarding the time period that had elapsed between the onset of symptoms and the first consultation when compared patients in self-medication group (11.46 months) with prescribed medication (14.6 months) (Mann-Whitney U=119.5; *p*=0.984).

## 4 DISCUSSION

Self-medication has been reported as an option people choose to relieve the suffering of conditions that cause pain. TMD can cause severe pain and affect the daily life of an individual, particularly while speaking and chewing. Thus, it is no surprise that these patients report to self-medication at times. However, the percentage of the population with TMD that self-medicate and how this behavior affects the severity of their condition in the first consultation remain unclear. The present study confirmed a high prevalence of self-medication, without any evidence that this behavior affects the severity of the condition.

Self-medication has social influence, access to medication directly on the shelves and overt marketing campaigns contribute to this type of behavior. Brazil is one of the world's painkillers consumer markets [10]. Chagas **et al*.* (2015) evaluated the use of analgesics in 145 patients with a headache diagnosed in Outpatient Headache Clinic. They found that 34% of patients performed self-medication [11]. Regarding analgesic used, drugs products with combinations of other substances with sodium dipyrone (32%) and sodium dipyrone (23%) were the most frequently used. These results contribute to results found in our study, that most of the patients reported self-medication used analgesics, particularly sodium dipyrone (n=12). The use of anti-inflammatories and analgesics is by no means contraindicated for patients suffering from pain caused by TMD. However, the use of these drugs should be based on clinical criteria, respecting the dosage, interval of administration and time of use that is recommended by a health professional. Elder **et al**. (2012) [6] in a study with 111 participants with TMD identified that 21 participants used opioids, 90 reported using NSAID, mainly ibuprofen, acetaminophen, naproxen and aspirin. These drugs have been incorporated into the dynamic of a consumer society, and as such, are subject to market interests that can be contrary to their main objective of prevention, diagnosis and treatment of illnesses.

Drug marketing trend to perpetuate ideals of normality and deservingness and offers the idioms of self-responsibility, self-care, and responsible citizenship they seek [7]. In our study, it was possible to observe a large time interval between the onset of symptoms and the demand for professionals, the use of self-medication and alleviation of symptoms due to easy access to medications can contribute for this situation. The seeking for a professional could be related to some factors such as pain intensity, pain acceptance, worsen of symptoms, fear of invasive treatments, treatment costs, lack of trained professional and treatment costs

Servidoni **et al*.* (2006) [12] assessed self-medication among ear, nose and throat patients. Questionnaires from 72 patients (mean age of 38 years) were assessed and the majority of the patients self-medicated (83%), although in 72% of these cases, they asked the clerk or pharmacist for advice. The most common types of drugs used were analgesics and antipyretics (90%), followed by flu medicine (78%) and anti-inflammatories (69%). In our study with patients with TMD, we found that 50% of patients report self-medication, this result may have been affected by the type and severity of symptoms, sociocultural profile of the participants and the society in which they live. Although both studies were performed in Brazil there may be differences regarding access to health services, access to information and the level of education of the participants. It is notable in our study that most of the patients who self-medicate have completed a third level course and do not require any help to choose the medication they use, probably due to their greater access to information. The questionnaire used could be improved and expanded to obtain data about how they search for information about the disease, as well as signs and symptoms and the therapeutic options available for the condition, on internet sites, social networks and in specialized magazines.

Different prevalence rates for TMD have been reported depending on the population studied. The gold standard for the diagnosis of TMD should be based on an assessment of the patient`s history as well as a clinical examination [13]. Several instruments have been created to assess TMD. The most commonly used tool is the RDC/TMD, which combines a clinical assessment and the medical history of the patient [14]. However, other instruments have been cited and used to assess the frequency of signs and symptoms, as well as the severity of TMD. The FAI has been proposed as a low cost and easy-to-apply alternative. In Brazil, the FAI has frequently been used to classify the severity of an individual’s TMD [9,15-17].

In the present study, the main pain complaint recorded was headache, followed by pain when opening the mouth and noises in the TMJ. TMD symptoms and headache are extremely common, the precise relationship between these two conditions is not known. A daily headache that occurs on awakening is often a symptom related to nocturnal bruxism. Many patients do not have an understanding about the disease and do not know the dentist as a professional of primary choice for pain in the face, head and neck. Thus, others health professionals usually were seeking, as shown in this study that 44.5% of patients sought other health professionals. This reflects the need to publicize the dentist as a qualified professional to diagnosis and treat myofascial and temporomandibular joint disorders. When the headache was not due to TMD, it is important to referral the patients to a neurologic evaluation to rule out other potential pathologic conditions [18]. Regarding otorrinolaringologista, two main factors that contribute to confusion between otologic pathology and TMD are the anatomic proximity and sensory innervation *via* auriculotemporal nerve. Patients often cannot differentiate ear pain from TMJ pain [18]. The challenge of each clinician is to be able to make an accurate diagnosis and referral the patient to the most appropriate specialist as a part of the multidisciplinary approach for TMD.

Zulqarnain **et al*.* (1998) [19] conducted a study to identify the symptoms correlated with TMD among female students. They analyzed the questionnaires of 705 students, with a mean age of 21.3 years. The authors confirmed the prevalence of fatigued jaws (34.5%), discomfort while chewing (31.3%), pain in the preauricular region (22.4%) and pain during maximal mouth opening (22.4%). Approximately 27.3% claimed to take medication, whether prescribed by a health professional or not. These authors presented an overview to an educational level, thus was not possible replicate these results for the population as a whole. In our study, no statistical difference was found between self-medication and the level of education (X^2^=0.810; *p*=0.667). This result could be related to the limited sample size, or by the fact that although there are different educational attainment, the knowledge about this disease is not available in an appropriate manner or people do not have access to this.

This was a preliminary study as it only involved one research center and a limited number of subjects. However, the aim of this research was to present a topic that should be explored using two easy-to-use and fast questionnaires. The limitations of the present study include the small size and heterogeneity of the sample and the absence of the patient´s clinical data.

## CONCLUSION

The idea of self-medicating before seeking professional help seems to be a common attitude among patients with TMD. However, for the sample studied and according to the Fonseca anamnestic index, this behavior was not correlated with the severity of the symptoms. The prevalence of self-medication seems not to be related to the time elapsed between the onset of symptoms and first consultation with a professional.

## Figures and Tables

**Chart 1:** Questions in the English language version of the Fonseca anamnestic Index according to Campos *et al*. (2009)^9^.

1. Do you have difficulty opening your mouth wide?
2. Do you have difficulty moving your jaw to the sides?
3. Do you feel fatigue or muscle pain when you chew?
4. Do you have frequent headaches?
5. Do you have neck pain or a stiff neck?
6. Do you have ear aches or pain in that area (TMJ)?
7. Have you ever noticed any noise in your TMJ while chewing or opening your mouth?
8. Do you have any habits such as clenching or grinding your teeth?
9. Do you feel that your teeth do not come together well?
10. Do you consider yourself a tense (nervous) person?

**Chart 2:** Questionnaire to assess the prevalence of self-medication among patients with temporomandibular disorder.

1.What is your main complaint, the reason for your appointment? 2.How long after your symptoms began did you seek professional help? 3.Who was the first person who contacted for help? ( ) Dentist ( ) Physiotherapist ( ) Speech Therapist ( ) Doctor – general clinic ( ) Doctor – Otolaryngologist ( ) Doctor – Neurologist ( ) Doctor – Other specialty: _________ ( ) Other: ______________________________
4.Have you taken any medication based on your own decision (without a prescription) before seeking this help? ( ) YES, which medicine? ( ) NO
5.When you took the medication you self-prescribed, did you ask anybody for help and if so, who? ( ) Pharmacist ( ) Clerk ( ) Friend/ Neighbor ( ) Relative ( ) Other __________________________________
6.Are you currently taking any medication? ( ) YES For what? Was it prescribed by a professional? ( ) YES ( ) NO

**Table 1:** Distribution of patients according to variables: Self-medication, gender, education level, age and time to seek professional help.

Self-medication	n*				Education level (n*)			Mean Age (years)	Mean Time (SD)**
					Primary School	High School	University		
Yes		17	Female	16	4	6	6	39.53	11.46 (13.84)
			Male	1	0	1	0		
No		17	Female	15	2	7	6	40	14.6 (15.02)
			Male	2	0	1	1		

* Number of patients ** The time period that elapsed between the onset of the symptoms and the first consultation in months.

## LIST OF ABBREVIATIONS

FAI: = Fonseca`s Anamnestic Index
TMD: = Temporomandibular Disorder
TMJ: = Temporomandibular Joint

## References

[1] Afolabi A.O., Akinmoladun V.I., Adebose I.J., Elekwachi G. (2010). Self-medication profile of dental patients in Ondo State, Nigeria.. Niger. J. Med..

[2] Klasser G.D., de Leeuw R. (2007). Medication use in a female orofacial pain population.. Oral Surg. Oral Med. Oral Pathol. Oral Radiol. Endod..

[3] Chaves T.C., De Oliveira A.S., Grossi D.B. (2008). Principais instrumentos para avaliação da disfunção temporomandibular, parte I : Indices e questionários; uma contribuição para a prática clínica e de pesquisa.. Fisioter. Pesqui..

[4] Liu F., Steinkeler A. (2013). Epidemiology, diagnosis, and treatment of temporomandibular disorders.. Dent. Clin. North Am..

[5] Klasser G.D., Greene C.S. (2009). Oral appliances in the management of temporomandibular disorders.. Oral Surg. Oral Med. Oral Pathol. Oral Radiol. Endod..

[6] Elder C., Ritenbaugh C., Aickin M., Hammerschlag R., Dworkin S., Mist S., Harris R.E. (2012). Reductions in pain medication use associated with traditional Chinese medicine for chronic pain.. Perm. J..

[7] Eaves E.R. (2015). “Just Advil”: Harm reduction and identity construction in the consumption of over-the-counter medication for chronic pain.. Soc. Sci. Med..

[8] Fonseca D., Bonfate G., Valle A., Freitas S. (1994). Diagnóstico pela anamnese da disfunção craniomandibular.. Rev. Gaucha Odontol..

[9] Campos J.A.B., Gonçalves D.A.G., Camparis C.M., Speciali J.G. (2009). Reliability of a questionnaire for diagnosing the severity of temporomandibular disorder.. Rev Bras Fisioter.

[10] Fraga P. (2013). As curvas da dor e do sorriso: Comercial de analgésicos substitui indicações médicas por valores do consumo e do trabalho para vender mais drágeas.. Dito Efeito.

[11] Chagas O.F., Éckeli F.D., Bigal M.E., Silva M.O., Speciali J.G. (2015). Study of the use of analgesics by patients with headache at a specialized outpatient clinic (ACEF).. Arq. Neuropsiquiatr..

[12] Servidoni A.B., Coelho L., Navarro M.L., Ávila F.G., Mezzalira R. (2006). Perfil da automedicação nos pacientes otorrinolaringológicos.. Rev. Bras. Otorrinolaringol..

[13] Mohl N.D. (1993). Reliability and validity of diagnostic modalities for temporomandibular disorders.. Adv. Dent. Res..

[14] Dworkin S.F., LeResche L. (1992). Research diagnostic criteria for temporomandibular disorders: Review, criteria, examinations and specifications, critique.. J. Craniomandib. Disord..

[15] Campos J.A.D.B., Carrascosa A.C., Bonafé F.S.S., Maroco J. (2014). Severity of temporomandibular disorders in women: Validity and reliability of the Fonseca Anamnestic Index.. Braz. Oral Res..

[16] Bevilaqua-Grossi D., Chaves T.C., de Oliveira A.S., Monteiro-Pedro V. (2006). Anamnestic index severity and signs and symptoms of TMD.. Cranio.

[17] Berni K.C., Dibai-Filho A.V., Rodrigues-Bigaton D. (2015). Accuracy of the Fonseca anamnestic index in the identification of myogenous temporomandibular disorder in female community cases.. J. Bodyw. Mov. Ther..

[18] Israel H.A., Davila L.J. (2014). The essential role of the otolaryngologist in the diagnosis and management of temporomandibular joint and chronic oral, head, and facial pain disorders.. Otolaryngol. Clin. North Am..

[19] Zulqarnain B.J., Khan N., Khattab S. (1998). Self-reported symptoms of temporomandibular dysfunction in a female university student population in Saudi Arabia.. J. Oral Rehabil..

